# Case Report: Atonic PNES Capture in fMRI

**DOI:** 10.3389/fneur.2022.803145

**Published:** 2022-02-21

**Authors:** Emmanuelle Hologne, Gabriela Hossu, Luca Fantin, Marc Braun, Cyril Husson, Louise Tyvaert, Coraline Hingray

**Affiliations:** ^1^Département de Neurologie, Centre Hospitalier Universitaire de Reims, Reims, France; ^2^IADI, U1254, Institut National de la Santé et de la Recherche Médicale et Université de Lorraine, Nancy, France; ^3^Centre d'Investigation Clinique-Innovation Technologique, Centre Hospitalier Régional Universitaire de Nancy, Nancy, France; ^4^Laboratoire Développement Adaptation Handicap (DevAH) EA 3450, Nancy, France; ^5^Département de Neuroradiologie Diagnostique et Thérapeutique, Centre Hospitalier Universitaire de Nancy, Nancy, France; ^6^Pôle Hospitalo-Universitaire de Psychiatrie d'Adultes et d'Addictologie du Grand Nancy, Centre Psychothérapique de Nancy, Laxou, France; ^7^Département de Neurologie, Centre Hospitalier Universitaire de Nancy, Nancy, France; ^8^Centre de Recherche en Automatique de Nancy, U7039, Centre National de la Recherche Scientifique et Université de Lorraine, Nancy, France

**Keywords:** functional imaging, functional neurological disorder, case report, conversion disorder, PNES, article types: case report

## Abstract

Psychogenic Non-Epileptic Seizures (PNES) are a misunderstood and disabling pathology, characterized by a paroxysmal occurrence of clinical signs without the epileptic activity. Resting-state functional MRI (fMRI) studies in patients with PNES have shown abnormal functional connectivity of the resting-state networks, especially in the limbic and motor systems, and in the precuneus. However, the transient nature of PNES episodes prevents us from elucidating the underlying mechanisms of seizures. Here, we report the case of a patient who presented an atonic episode of PNES during a 3T fMRI session. The patient is a 23-year-old woman, suffering from post-traumatic stress disorder, with no neurological comorbidities. The preprocessing of the fMRI images involved realignment, co-registration, segmentation, normalization, denoising (PhysIO toolbox), and smoothing. The time boundary of the seizure was defined according to the patient's reports, and the seizure period was contrasted with the resting state period before the seizure. A whole-brain analysis showed significant activations (left inferior temporal gyrus, left temporo-occipital junction) and deactivations (right precuneus, right superior parietal lobule, right postcentral gyrus, bilateral lingual gyri, inferior occipital gyri, and cerebellar lobules; right insula in a sub-thresholded analysis). Activations and deactivations occurred in four cerebral networks: emotional processing, agency, self-perception, and dissociation. To our knowledge, this report is the first published case of functional MRI during PNES. These results could confirm the emotional and dissociative hypothesis of the physiopathology of PNES and highlight future targets for neuromodulation.

## Introduction

Psychogenic non-epileptic seizures (PNES) are a frequent and disabling pathology (1) with a substantial societal cost (2). They are characterized by a paroxysmal occurrence of clinical signs without a visible epileptic activity on electroencephalography (EEG) (3). However, although its clinical presentation is wellknown, its physiopathology remains enigmatic. The PNES are probably a dissociative and emotional pathology. Indeed, emotional processing in PNES is probably different from a healthy state, and overwhelming emotions must be suppressed. The PNES would be the result of an excessive suppression or an emotional inhibition of this emotion (4). However, this emotional inhibition could provoke side effects such as dissociation, defined as the disruption and/or discontinuity in the normal integration of consciousness, memory, identity, emotion, perception, body representation, motor control, and behavior (according to the Diagnostic and Statistical Manual of Mental Disorders Fifth Edition).

Because PNES are unpredictable and transient, two types of studies investigating the PNES physiopathology were performed: ictal (during the seizure) and interictal studies (between seizures). Regarding ictal studies, one work has shown a decreased functional connectivity between the anterior insula, hippocampus, amygdala, parietal, and prefrontal cortices during PNES (5). This work used a stereo-electroencephalography in two patients with epilepsy, which is a technology providing high temporal resolution but a low spatial sampling. Thanks to an excellent spatial resolution of the whole brain, functional MRI (fMRI) can supply evidence of brain areas and networks involved in this pathology. Therefore, inter-ictal studies have used neuroimaging to investigate resting-state networks in patients with PNES. The findings of these studies have shown that the precuneus involved in dissociation (6) was rather involved in the default mode network (DMN) in patients with PNES (7), and the insula, known to be involved in the neural network of emotional processing, was more connected to the sensorimotor network (8). These results suggest that the resting network in patients with PNES is different for the healthy ones, with a higher tendency to dissociation and high connection between the emotional process and motor behavior. This provides evidence of the proposed model of PNES physiopathology (4). However, these works have a strong limitation, as they used a resting-state methodology, during which there are neither emotional nor dissociative tasks, nor clinical signs. Therefore, brain mechanisms occurring during seizures remain unknown, preventing the emergence of neuro-modulating targeted treatments.

Here, we report the first case of a patient who presented a spontaneous episode of PNES recorded in 3-Tesla (3T) fMRI. This analysis allows us, at least a little, to unravel the mysteries of PNES.

## Case Description

### Patient

The patient is a 23-year-old right-handed woman. She presents a post-traumatic stress disorder with reviviscences linked to physical and emotional abuse during childhood and adolescence. The diagnosis of PNES was clinically established by an experienced epileptologist according to the ILAE report (3). Her PNES began at 16 years old, and she is used to presenting around 4 episodes of PNES per month. Her seizures are atonic (9); she presents a global hypotonia and, sometimes, few clonic movements of limbs. A few moments before the hypotonia, she feels an undefined prodromal sensation that prevents her. Then, she describes a feeling of “blocking” with an absolute impossibility to voluntarily move and without alteration of consciousness. Interictal physical examination was normal. She has no further medical history, specifically no epilepsy or cranial injury, and no other psychiatric comorbidity. She takes no psychiatric or neurological drugs and receives cognitive-behavioral therapy and hypnotherapy at the psychiatric department of the University.

### fMRI Acquisition

Whole-brain functional MR images were acquired using a 3T magnet (Magnetom Prisma; Siemens Healthcare GmbH, Germany) with a 64-channel receive-only head coil. High-resolution anatomical images were acquired using a 3D Turbo Flash sequence (TR = 2,200 ms; TE = 2.93 ms; TI = 900 ms; flip angle = 8°; in plane resolution =0.90 × 0.90 mm2; slice thickness = 1 mm). 160 volumes parallel to the anterior–posterior commissural line were acquired, covering the whole brain. Functional images were acquired using a GE-EPI sequence (TR = 2,000 ms; TE = 30 ms; 48 axial slices; slice thickness = 2.5 mm; slices order: interleaved; in plane resolution = 1.97 × 1.97 mm^2^; flip angle = 77°).

The patient gave free and informed written consent to participate in the study Emotional Lived in Patients Suffering From Psychogenic Non-epileptic Seizures (EMOCRISES) (NCT02976545). We asked the patient to keep her eyes closed during the resting-state session of 8 min and 40 s, without falling asleep, and to stay still during the acquisition. In the context of EMOCRISES, the patient had an answer box in her right hand to interact during the experimental session. In this case, the patient used the answer box to inform the staff of the beginning and the end of her seizure.

### MRI Data Analysis

Images were preprocessed using SPM12 (Statistical Parametric Mapping, Version 12, Revision 7219, Welcome Department of Cognitive Neurology, London, UK, RRID: SCR_007037) on Matlab [version 7.10.0 (R2010a). Natick, Massachusetts: The MathWorks Inc.; 2010, RRID: SCR_001622]. To optimize the movement correction, we applied the first co-registration between each functional image and the anatomical image. We then applied a standard preprocessing (realignment, co-registration, normalization, and smoothing). We used TAPAS PhysIO Toolbox (The PhysIO Toolbox for Modeling Physiological Noise in fMRI Data) (10) to denoise functional volumes of white matter and a cerebrospinal fluid signal.

We defined the control period from the 20 s after the beginning of the resting-state session to 20 s before the beginning of the seizure (total duration: 60 s). We defined the seizure period as the period between the two moments of patient reports. Using an SPM 12, we modeled the GE-EPI time series using a first-level general linear model (11), with regressors representing the expected BOLD fMRI response to seizure and to rest according to a block-paradigm and hemodynamic response function (HRF). Realignment parameters and denoising regressors were added to the regression models. Finally, we used a 128-s-high-pass filter to remove the non-physiological slow signal shifts.

The T-maps were calculated between rest and seizure. Results were visualized using the BSPMVIEW toolbox for SPM (10.5281/zenodo.168074).

### Statistical Analysis

We performed statistical analyses in the whole brain and in regions of interest (ROI) (using Neuromorphometrics atlas). We explored both activations and deactivations. The T-maps were thresholded to obtain an uncorrected *p* < 0.001, and we applied a spatial extent threshold on the activated clusters to obtain *p* < 0.05, corrected for the multiple comparisons (the FWE method) (11). We also performed a sub-thresholded analysis by decreasing T-threshold, to explore other areas eventually involved in PNES mechanisms.

## Diagnostic Assessment: fMRI Results

### Seizure Features

The subject presented one typical PNES event during the fMRI resting-state session. The atonic seizure lasted 220s.

### Whole-Brain Analysis

**Figure 1:**
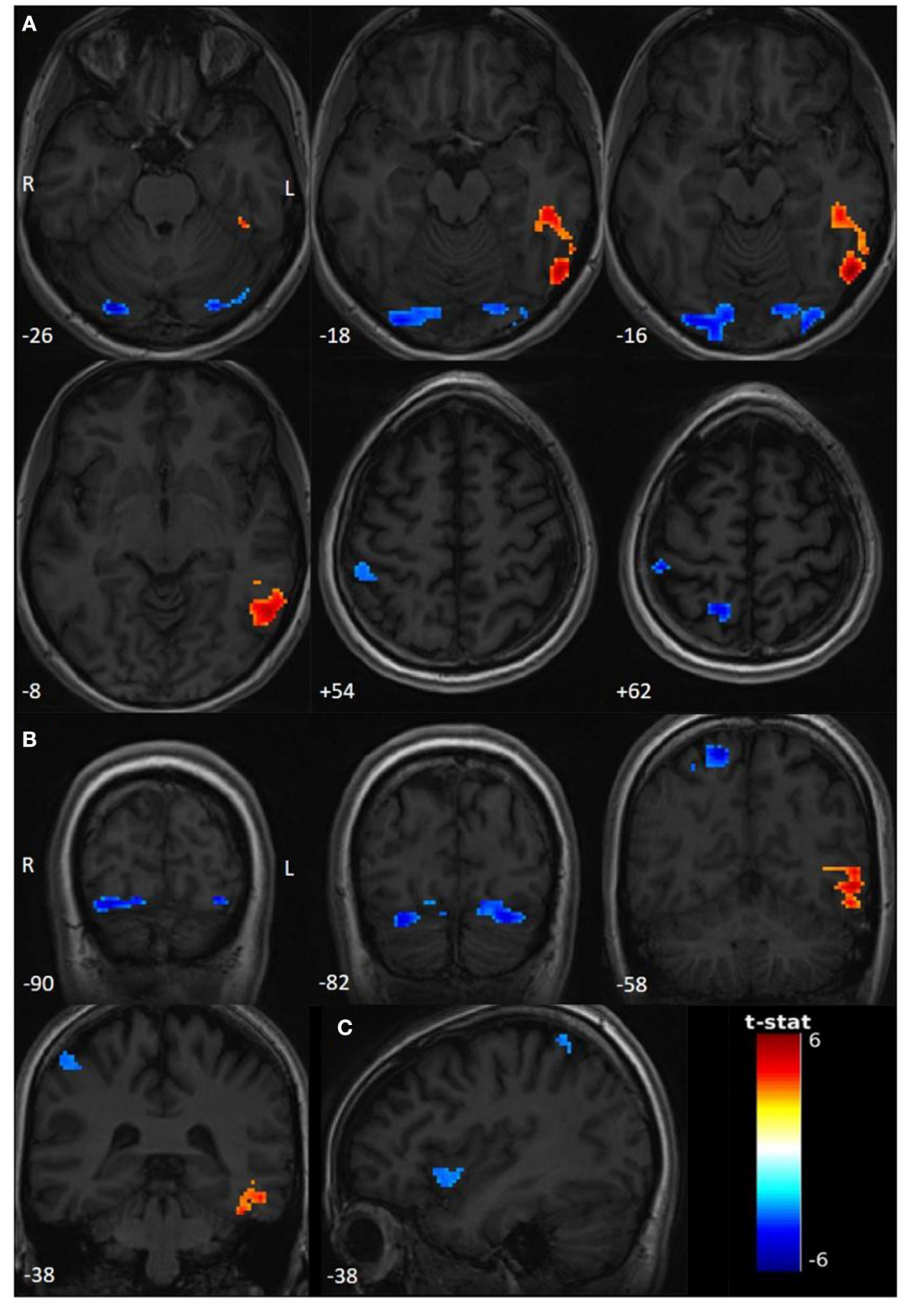
Functional activations and deactivations during contrast PNES > rest (*p* uncorr < 0.001; cluster-level FWE *p* < 0.05; neuroradiological convention). The numbers on the bottom left correspond to the MNI coordinates [**(A)** Z coordinate, **(B)** Y coordinate, and **(C)** X coordinate]. The letters L and R correspond respectively to the left and right sides. The palette in warm tones corresponds to activations and the one in cold tones to deactivations. The color bar corresponds to T-scores. **(A)** Significant activations and deactivations for *T* > 3.1595, axial slices. **(B)** Significant activations and deactivations for *T* > 3.1595, coronal slices. **(C)** Sub-thresholded deactivations of right insula for *T* > 3.1.

Compared to the rest period, we found significant activations (*T* > 3.1595, *p* < 0.001) in the left inferior temporal gyrus (ITG) and the left temporo-occipital junction (TOJ) during the seizure period. Deactivations involved the right precuneus, the right superior parietal lobule (SPL), and the right postcentral gyrus (PoG), bilateral lingual gyri, bilateral inferior occipital gyri (IOG), and bilateral cerebellar lobule (Crus 1). Interestingly, using a sub-threshold analysis (T > 3.1; *p* < 0.001), we observed an additional deactivation in the anterior right insula (Figure 1, Table 1).

**Table 1:** Anatomical localization of the significant clusters.

	**Area**	**MNI coordinates**			**Cluster size**	***T*** **-value**
	**Positive**	**X**	**Y**	**Z**		
Left	Temporoccipitaljunction	−54	−64	−16	573	6,4
	Inferior temporal gyrus	−48	−34	−18		4,95
	*Negative*					
Left	Crus 1	−26	−82	−24	200	−4,95
	Inferior occipital gyrus	−30	−90	−14		−4,65
	lingual gyrus	−14	−84	−16		−4,26
Right	Crus 1	26	−84	−24	301	−6,6
	lingual gyrus	30	−90	−16		−5,1
	Inferior occipital gyrus	20	−98	−14		−4,42
	Precuneus	16	−58	−64	149	−5,62
	Post central	48	−34	−62	106	−4,68

### ROI Analysis

Regarding the emotional hypothesis of PNES mechanisms, we defined ROIs as regions involved in emotional processing: the anterior cingular cortex, and both the insula and amygdala bilaterally. No significant deactivations or activations were found.

## Discussion

This is, according to our knowledge, the first report of functional MRI of a spontaneous PNES. Significant brain activations (left ITG; left TOJ) and deactivations (right insula, right precuneus, right SPL, right PoG, bilateral lingual gyri, IOG, and cerebellar lobule) were observed during PNES in contrast to a resting period immediately preceding the seizure. Our data had high quality. The recorded seizure was atonic, which gives low-motion artifacts, and, unlike SEEG, fMRI allows for whole-brain analyses. This quality allows us to make assumptions about activations and deactivations during this type of seizure. It seems to involve specific networks: emotional processing, dissociation, self-perception, and sense of agency.

### Emotional Network

We showed a deactivation in the right anterior insula, strongly related to emotional processing (12). The left ITG, involved in the ventral visual pathway, could be also involved in emotional regulation (13), like bilateral lingual gyri (14). Moreover, bilateral Crus 1 is involved in multisensory emotional processing (15).

### Dissociation

We found out deactivation in the precuneus, which is involved in dissociation (6), that is to assay a disconnection between the mind and the body. As already described above, patients with PNES had a higher involvement of the precuneus in the DMN than healthy controls. Moreover, this involvement was correlated with dissociation scores (7).

### Body Ownership (BO) and Peripersonal Space (PPS)

We showed a deactivation in the right PoG and SPL and an activation in the left TOJ. The BO is the faculty to recognize one's own body as being part of oneself. The PPS is a neuronal network, where each neuron is able to encode multisensory inputs in a receptive field localized close to the body. These two concepts lead to self-construction and self-perception. The right PoG and SPL are involved in these processes (16, 17). The left TOJ is a highly associative region (notably related to vestibular illusions), and it could be involved in BO (16).

### Agency

The right PoG, right SPL, and left TOJ could be also involved in the self-agency network. On the one hand, self-agency is the ability to recognize oneself as the agent of one's actions. This network involves the associative motor cortices and the primary and associative sensory cortices like the right PoG and SPL (18). On the other hand, a negative agency is the experience of reduced control of motility, and the left TOJ cluster is spatially close to this network (19).

Our work has limitations. Indeed, we only recorded one “declared” PNES, and we do not know if the activation/deactivation pattern is reproducible. Moreover, it could be surprising to observe that there is no involvement of the motor network, especially, given the atonic type of PNES in this patient. However, our analysis was performed on the global timing of the PNES to increase the signal-to-noise ratio, and we did not analyze the dynamic BOLD signal changes over the duration of the PNES. Motor networks could be involved, but in a time-limited manner, which is a lead that was not explored here. Furthermore, the rest period defined just before PNES may not be fully occupied by rest, as the brain activity could change before the subject's perception of the onset of her seizure. This rest period could also be noisy by voluntary motor action of the use of the press button. Moreover, ROI analyses may not have demonstrated any significant responses due to the weak power of our work based on a single case. Finally, these involved areas are highly associative regions involved in other networks, and our interpretation could be disputable. However, given the PNES pathology and the semiology of our patient, this interpretation seems, to us, to be the more relevant.

To summarize, body scheme, agency, emotional processing, and dissociation appear to be involved in PNES. However, none of these networks have been completely activated or deactivated, as if PNES were not the result of activation or deactivation of one or more welldefined networks but, rather, as an intrinsic imbalance of these networks. This pattern highlights the fundamental role of self-perception and emotional processing during PNES, as it has been suggested for years (4). This work ought to be replicated on larger samples in order to improve our understanding of the physiopathology of PNES, which will lead to precise targets for neuromodulation and more specific treatment.

## Data Availability Statement

The raw data supporting the conclusions of this article will be made available by the authors, without undue reservation.

## Ethics Statement

The studies involving human participants were reviewed and approved by Comité de protection des personnes. The patients/participants provided their written informed consent to participate in this study.

## Author Contributions

GH, CHu, CHi, and LT conceived the experiment. GH, CHi, and MB performed the experiment. EH, GH, CHi, and LT analyzed the data. EH wrote the manuscript in concertation with LF, GH, MB, CHu, CHi, and LT. All authors contributed to the article and approved the submitted version.

## Funding

This study was supported by the Nancy University Hospital, and a grant was provided by the French Ministry of Health (APJ 2015).

## Conflict of Interest

The authors declare that the research was conducted in the absence of any commercial or financial relationships that could be construed as a potential conflict of interest.

## Publisher's Note

All claims expressed in this article are solely those of the authors and do not necessarily represent those of their affiliated organizations, or those of the publisher, the editors and the reviewers. Any product that may be evaluated in this article, or claim that may be made by its manufacturer, is not guaranteed or endorsed by the publisher.
